# Supplementary material for: A pivot-tether model for nucleosome recognition by the chromosomal passenger complex
Source: EMBO Rep. 2025 Jul 15;26(17):4219–47. doi: 10.1038/s44319-025-00523-4 (PMC12420818; doi:10.1038/s44319-025-00523-4)
Supplement: Supplementary file 1 — Appendix [file 44319_2025_523_MOESM1_ESM.pdf]

Appendix to:

A pivot-tether model for nucleosome recognition by the chromosomal passenger complex

Reinis R. Ruza, Chyi Wei Chung, Danny B.H. Gold, Michela Serena, Emile Roberts, Ulrike Gruneberg, and Francis A. Barr

Page 2. Appendix Figure S1. Comparison of Aurora B and Sgo1 localisation to the pericentromere.
Page 3. Appendix Figure S2. Analysis of individual CPC subunits.
Page 4. Appendix Figure S3. Production and characterisation of the CPC and H3pT3-nucleosomes.
Page 5. Appendix Figure S4. Defining a minimal borealin fragment for centromere targeting.
Page 6. Appendix Figure S5. Cryo-EM data processing of CPC-nucleosome complexes.

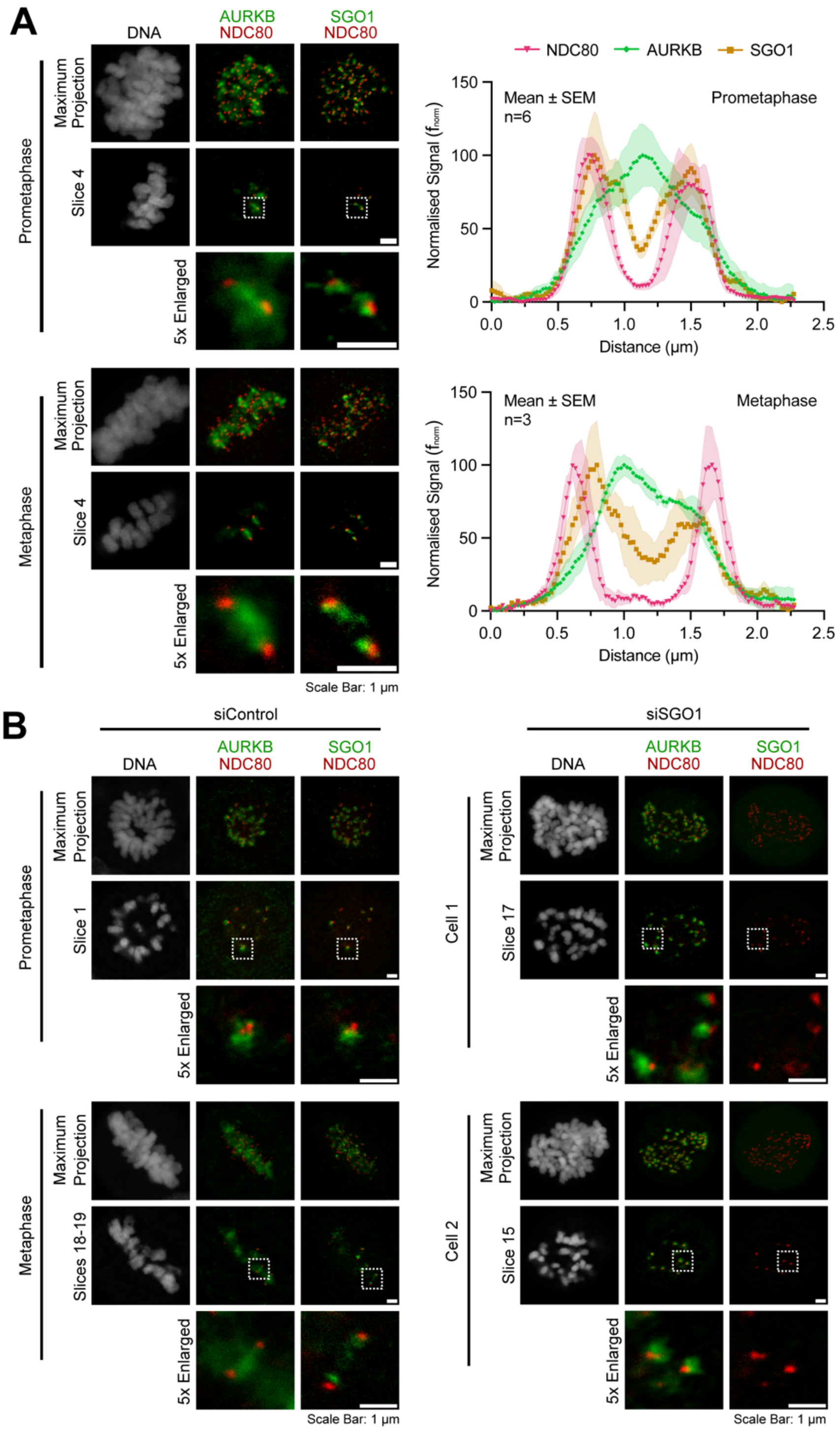

Appendix Figure S1. Comparison of Aurora B and Sgo1 localisation to the pericentromere.
(A) STED images depicting NDC80, Sgo1 and Aurora B (AURKB) in prometaphase and metaphase HCT116 NDC80-HaloTag cells. DNA was stained with Picogreen. Representative maximum intensity projections and selected slices are shown. Line scans show signal intensity across kinetochore pairs (mean ± SEM, sample size n = 6 or n = 3 indicated in figure). (B) HCT116 NDC80-HaloTag cells depleted of Sgo1 for 72 h using siRNA (siSgo1) or a control (siControl) were imaged using STED microscopy. Representative images show NDC80, Sgo1 and Aurora B (AURKB) in prometaphase and metaphase for the control condition. Because Sgo1 depleted cells lose sister chromatid cohesion, two representative cells are shown in a prometaphase-like state.

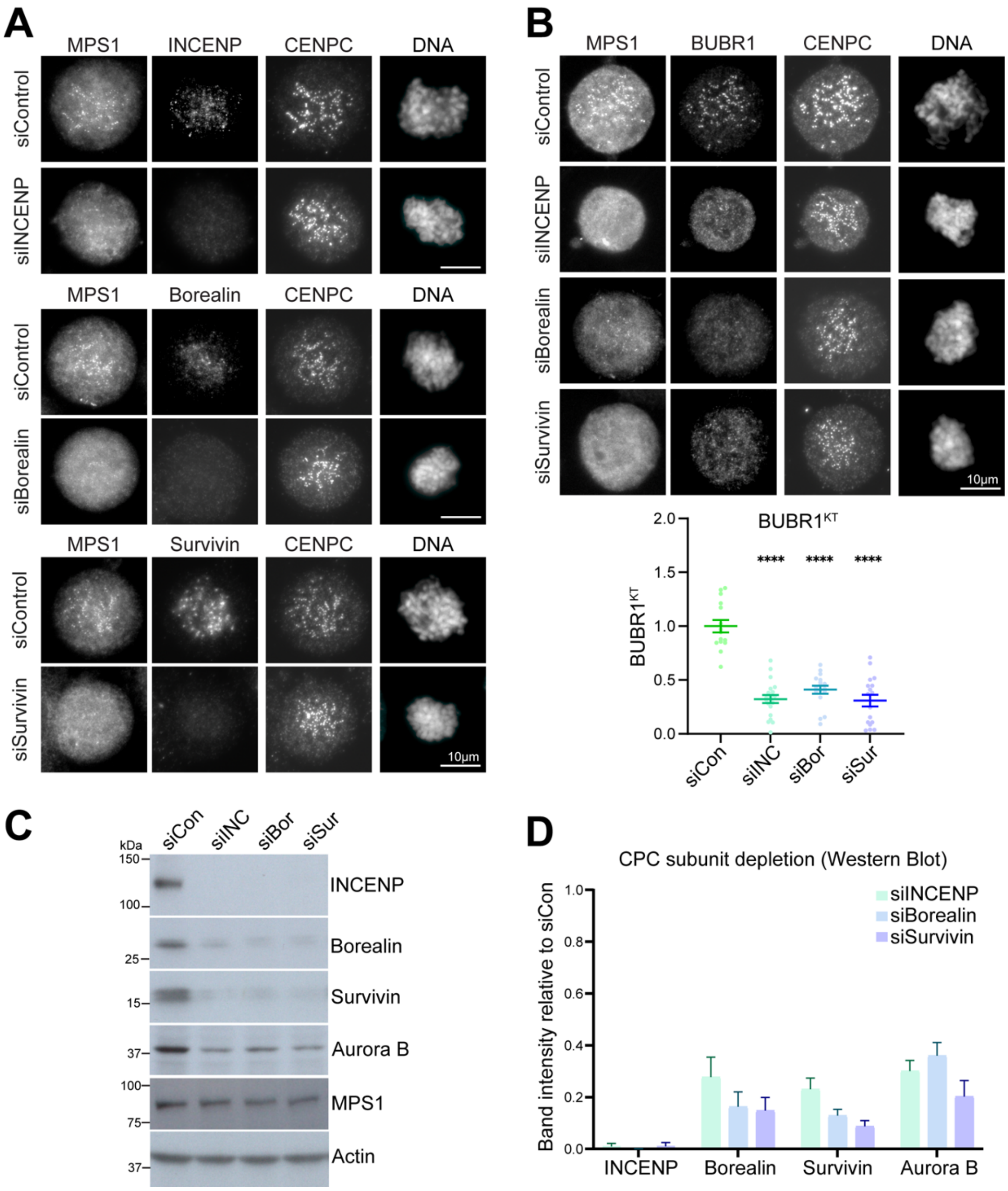

Appendix Figure S2. Analysis of individual CPC subunits.
(A) Dependence of MPS1 and (B) BUBR1 kinetochore localisation on INCENP (ICP), borealin (Bor), or survivin (Sur) was tested using siRNA depletion in prometaphase-arrested HeLa MPS1-GFP cells. Representative images with a scale bar of 10 µm are shown. A scatter plot with mean ± SEM for BUBR1^{KT} and a Kruskal-Wallis test and Dunn's test for multiple comparisons for significance to the control (siCon), p<0.0001 (****) and p<0.001 (***). Sample sizes were n>15 cells from at least 3 independent experiments. (C) Western blot of CPC subunit depletion compared to control and (D) mean CPC subunit levels after 48 h (mean ± SD, n=3 independent experiments).

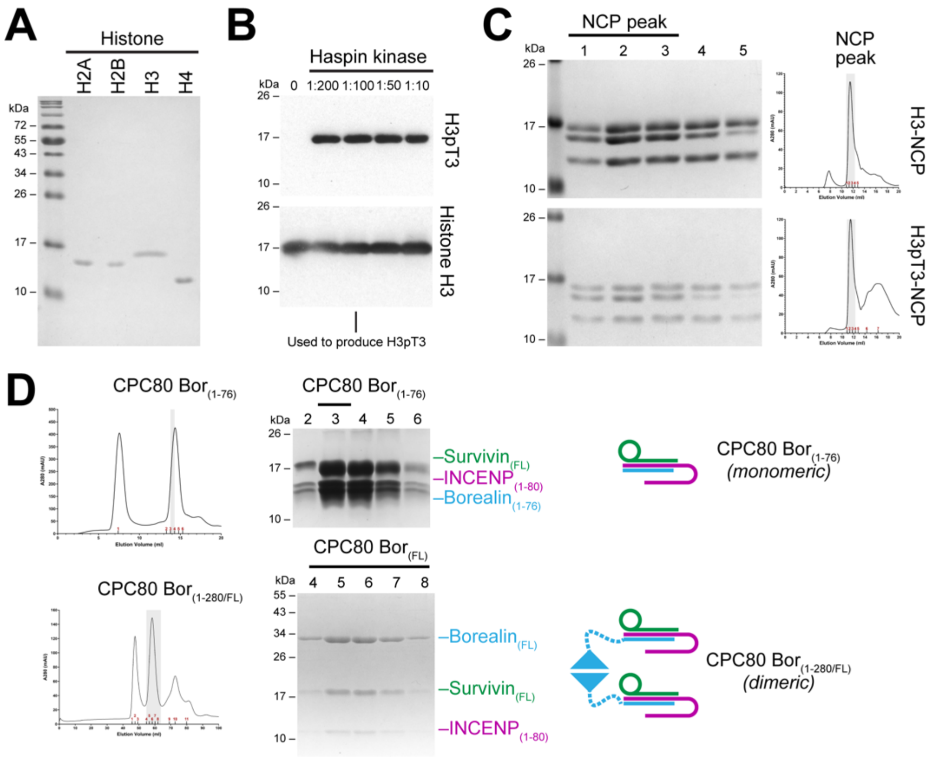

Appendix Figure S3. Production and characterisation of the CPC and H3pT3-nucleosomes.
(A) Purified histones used for nucleosome assembly. (B) Recombinant haspin kinase phosphorylation of histone H3 at the specific molar ratios. For large-scale preparation, a 1:100 ratio of haspin kinase to histone H3 was used. (C) Size exclusion chromatography of assembled nucleosome core particles using haspin-phosphorylated (H3pT3-NCP) or non-phosphorylated histone H3 (H3-NCP). (D) Purified CPC subcomplexes containing the indicated full-length (FL) or truncated (numbers indicating start-end amino acids).

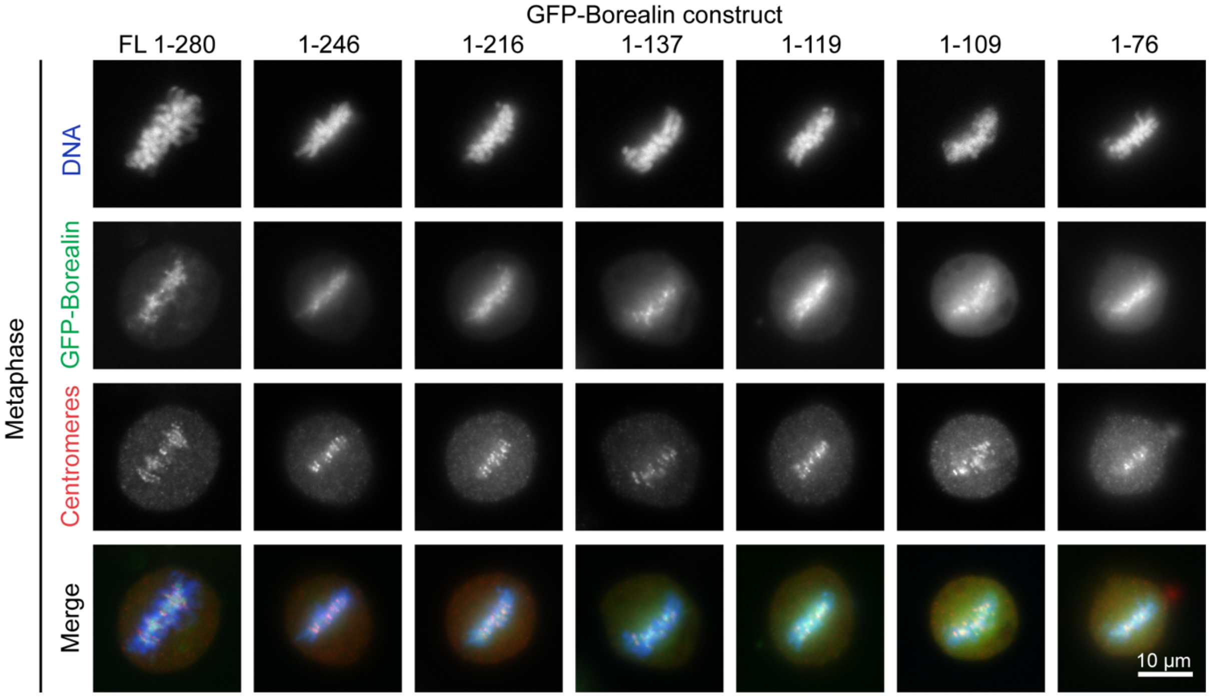

Appendix Figure S4. Defining a minimal borealin fragment for centromere targeting.
HeLa cells were transfected with the indicated GFP-borealin truncation constructs for 24 h and then fixed and processed for microscopy. Centromeres were stained with anti-centromere antibodies (predominantly CENP-A/B/C). DNA was detected using Hoechst-33528. Single images are shown representative of multiple independent experiments. Scale bar marks 10 µm.

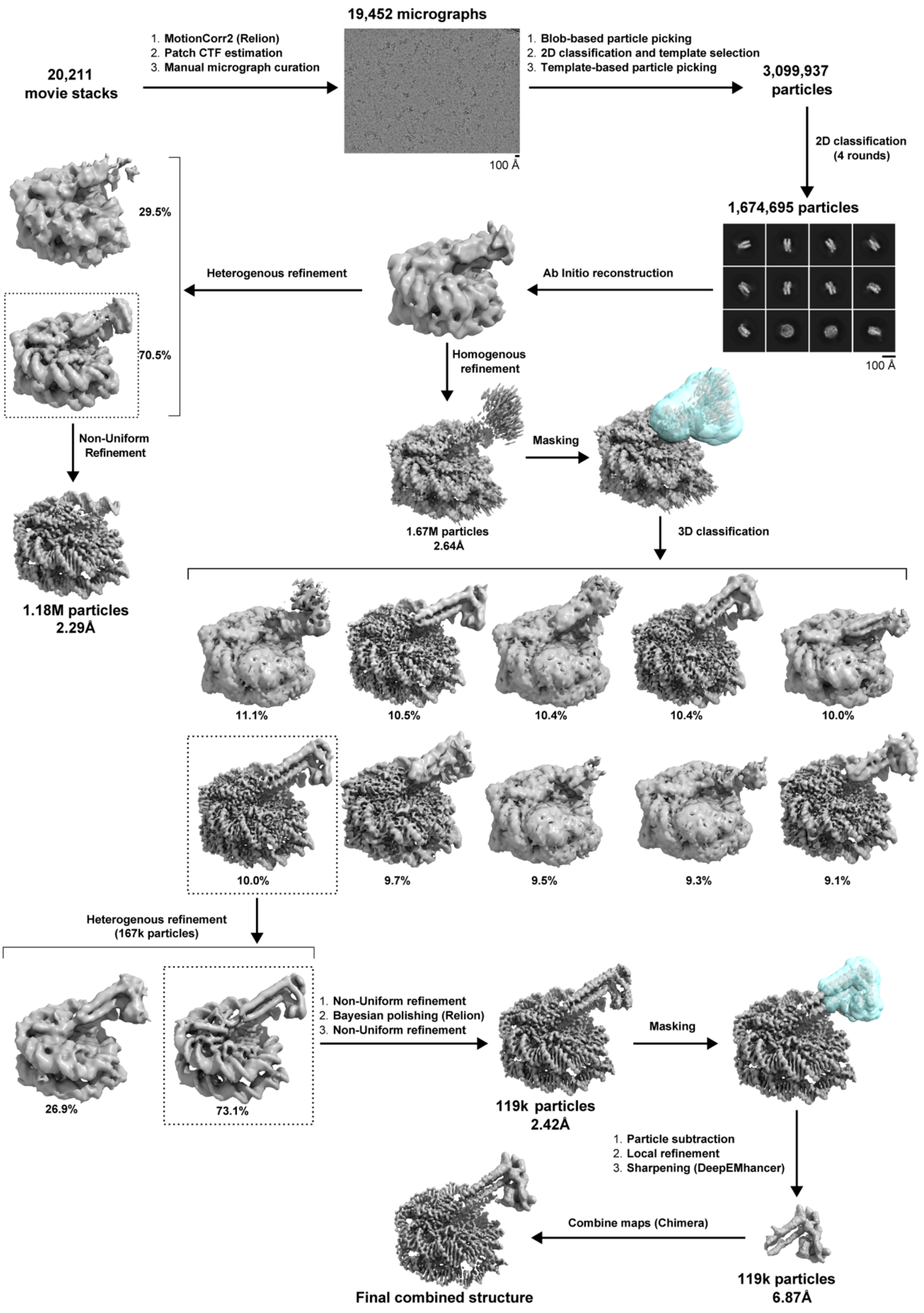

Appendix Figure S5. Cryo-EM data processing of CPC-nucleosome complexes.
Grey blobs indicate resulting EM maps. Percentages indicate the share of the particles belonging to a specific class. Dotted boxes indicate classes selected for further processing. Global resolution values given based on Fourier shell correlation gold standard value (0.143). Masks are indicated in cyan. Unless otherwise specified, all data processing was performed in CryoSparc v4.4.0.
