# Supplementary material for: A pivot-tether model for nucleosome recognition by the chromosomal passenger complex
Source: EMBO Rep. 2025 Jul 15;26(17):4219–47. doi: 10.1038/s44319-025-00523-4 (PMC12420818; doi:10.1038/s44319-025-00523-4)
Supplement: Supplementary file 2 — Table EV1 [file 44319_2025_523_MOESM2_ESM.pdf]

	pNCP-CPC80$_{(Borealin-FL)}$, BS3-crosslinked	pNCP	pNCP, BS3-crosslinked	**A.** pNCP-CPC80$_{(Borealin\ 1-76)}$, all particles	**B.** pNCP-CPC80$_{(Borealin\ 1-76)}$, after 3D classification	**C.** pNCP-CPC80$_{(Borealin\ 1-76)}$, CPC80 distal end local refinement	**D.** pNCP-CPC80$_{(Borealin\ 1-76)}$, final combined structure
Data Collection							
Microscope	FEI Titan Krios	Talos Arctica	Talos Arctica	FEI Titan Krios	FEI Titan Krios	FEI Titan Krios	FEI Titan Krios
Camera	Gatan K3	TFS Falcon 4	TFS Falcon 4	Gatan K3	Gatan K3	Gatan K3	Gatan K3
Magnification	64,000x	92,000x	120,000x	105,000 x	105,000 x	105,000 x	105,000 x
Voltage (kV)	300	200	200	300	300	300	300
Electron dose ($e^-/Å^2$)	33.80	28.94	31.06	43.86	43.86	43.86	43.86
Defocus range (µm)	-1.0 to -2.3	-1.2 to -3.0	-1.2 to -3.0	-1.0 to -2.3	-1.0 to -2.3	-1.0 to -2.3	-1.0 to -2.3
Collection Mode	2x-Binned Super-Resolution	Counting	Counting	2x-Binned Super-Resolution	2x-Binned Super-Resolution	2x-Binned Super-Resolution	2x-Binned Super-Resolution
Pixel size (Å)	1.4	1.5	1.2	0.83	0.83	0.83	0.83
Movies	3,042	2,278	3,222	20,239	20,239	20,239	20,239
Initial particles	1,287,276	491,041	1,051,059	3,099,937	3,099,937	3,099,937	3,099,937
Final particles	146,173	115,357	102,960	1,180,547	119,063	119,062	119,063
Model Composition							
Proteins present	-	-	-	H2.A, H2.B, H3, H4, Borealin N-terminus	H2.A, H2.B, H3, H4, Borealin, Survivin, INCENP	Borealin, Survivin, INCENP	H2.A, H2.B, H3, H4, Borealin, Survivin, INCENP
Non-hydrogen atoms	-	-	-	12,147	13,968	1,789	14,025
Protein residues	-	-	-	784	1014	224	1,021
DNA bases	-	-	-	147	147	-	147
Refinement							
Resolution (Å)	-	-	-	2.3	2.4	6.9	-
CC							
$CC_{(mask)}$	-	-	-	0.88	0.85	0.71	0.71
$CC_{(volume)}$	-	-	-	0.88	0.84	0.71	0.71
$CC_{(NCP)}$	-	-	-	0.89	0.88	-	0.82
$CC_{(CPC)}$	-	-	-	0.72	0.61	0.71	0.60
Average B factor ($Å^2$)							
Protein	-	-	-	66.48	54.66	104.23	59.74
DNA	-	-	-	55.52	42.39	-	42.39
R. m. s. deviations							
Bond lengths (Å)	-	-	-	0.004	0.006	0.007	0.006
Bond angles (º)	-	-	-	0.573	0.860	1.256	0.864
Validation							
MolProbity Score	-	-	-	1.01	1.32	1.95	1.37
Clashscore	-	-	-	2.34	5.80	28.97	6.79
Poor rotamers (%)	-	-	-	0.64	0.61	0.00	0.61
Ramachandran plot							
Favoured (%)	-	-	-	98.30	98.08	98.59	98.09
Allowed (%)	-	-	-	1.70	1.92	1.41	1.91
Disallowed (%)	-	-	-	0.00	0.00	0.00	0.00

Table 1. Cryo-EM data collection and model refinement information. pNCP - NCP-H3.T3p; Borealin - Borealin$_{(1-76)}$; INCENP - INCENP$_{(1-80)}$; Survivin - Survivin$_{(FL)}$, Borealin N-terminus - Borealin$_{(1-23)}$. Model refinement performed using Phenix 1.20.1 and validation statistics obtained by MolProbity.
