# Supplementary material for: A pivot-tether model for nucleosome recognition by the chromosomal passenger complex
Source: EMBO Rep. 2025 Jul 15;26(17):4219–47. doi: 10.1038/s44319-025-00523-4 (PMC12420818; doi:10.1038/s44319-025-00523-4)
Supplement: Supplementary file 4 — Expanded View Figures [file 44319_2025_523_MOESM4_ESM.pdf]

Expanded View Figures

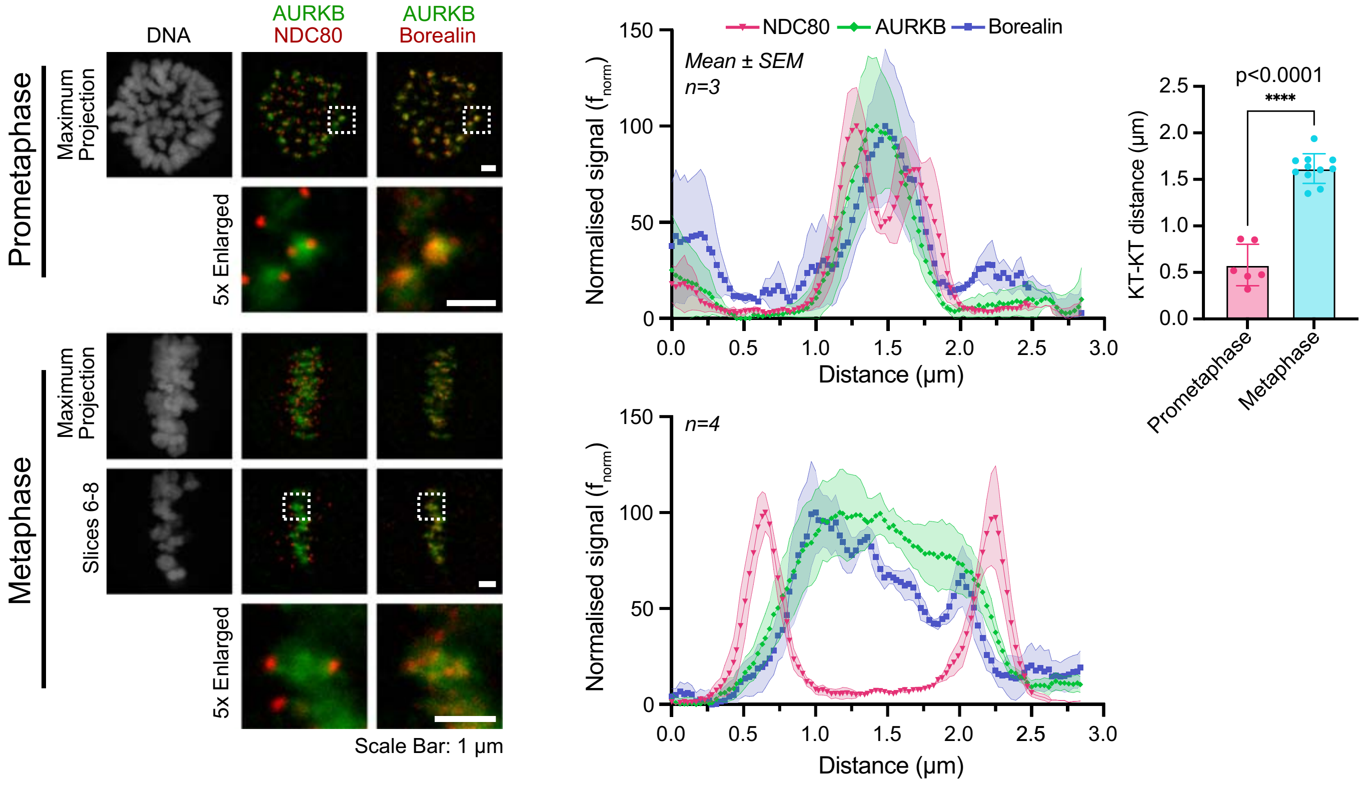

Figure EV1. The outer kinetochore and CPC^{borealin} are spatially separated in metaphase.

STED images depicting NDC80, borealin and Aurora B (AURKB) localisation in prometaphase and metaphase HCT116 NDC80-HaloTag cells. DNA was stained with Picogreen. Representative maximum intensity projections and selected slices are shown (mean ± SEM, sample size $n = 3$ or 4 as indicated in the figure). Line scans show signal intensity across kinetochore pairs. Bar graphs show mean kinetochore-kinetochore (KT-KT) distances in prometaphase and metaphase (mean ± SD). For KT-KT distances prometaphase $n = 6$, metaphase $n = 11$ with a two-tailed unpaired t test, $P < 0.0001$ (****).

© The Author(s)

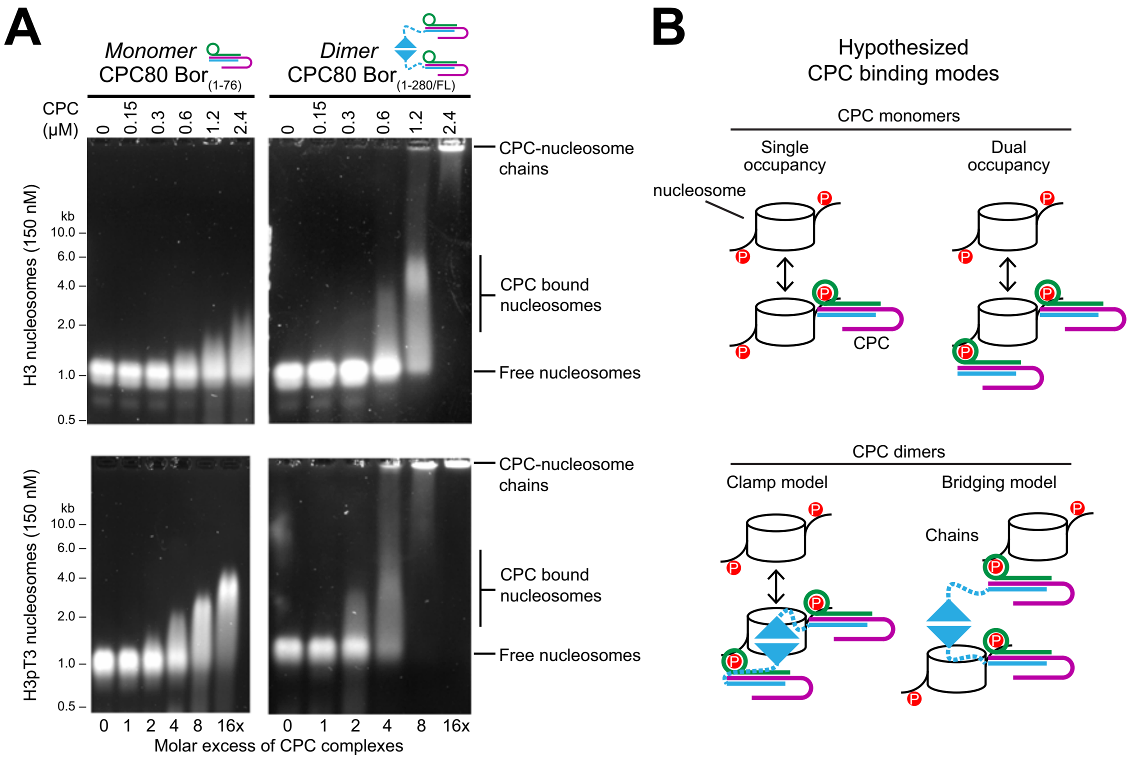

Figure EV2. Interaction of monomeric and dimeric CPC complexes with H3pT3 nucleosomes.

(**A**) EMSAs between 150 nM H3 T3-phosphorylated (H3pT3) nucleosomes or non-phosphorylated H3-nucleosomes and CPC80-Bor$_{(1\text{-}76)}$ or CPC80-Bor$_{(FL)}$ targeting module complexes at the specified molar excesses. Predicted species are indicated on the right of the gel panels. (**B**) A schematic depicting hypothesized binding modes for monomeric and dimeric forms of the CPC and histone H3pT3 nucleosomes (survivin in green; borealin in light blue, INCENP in magenta).

 © The Author(s)

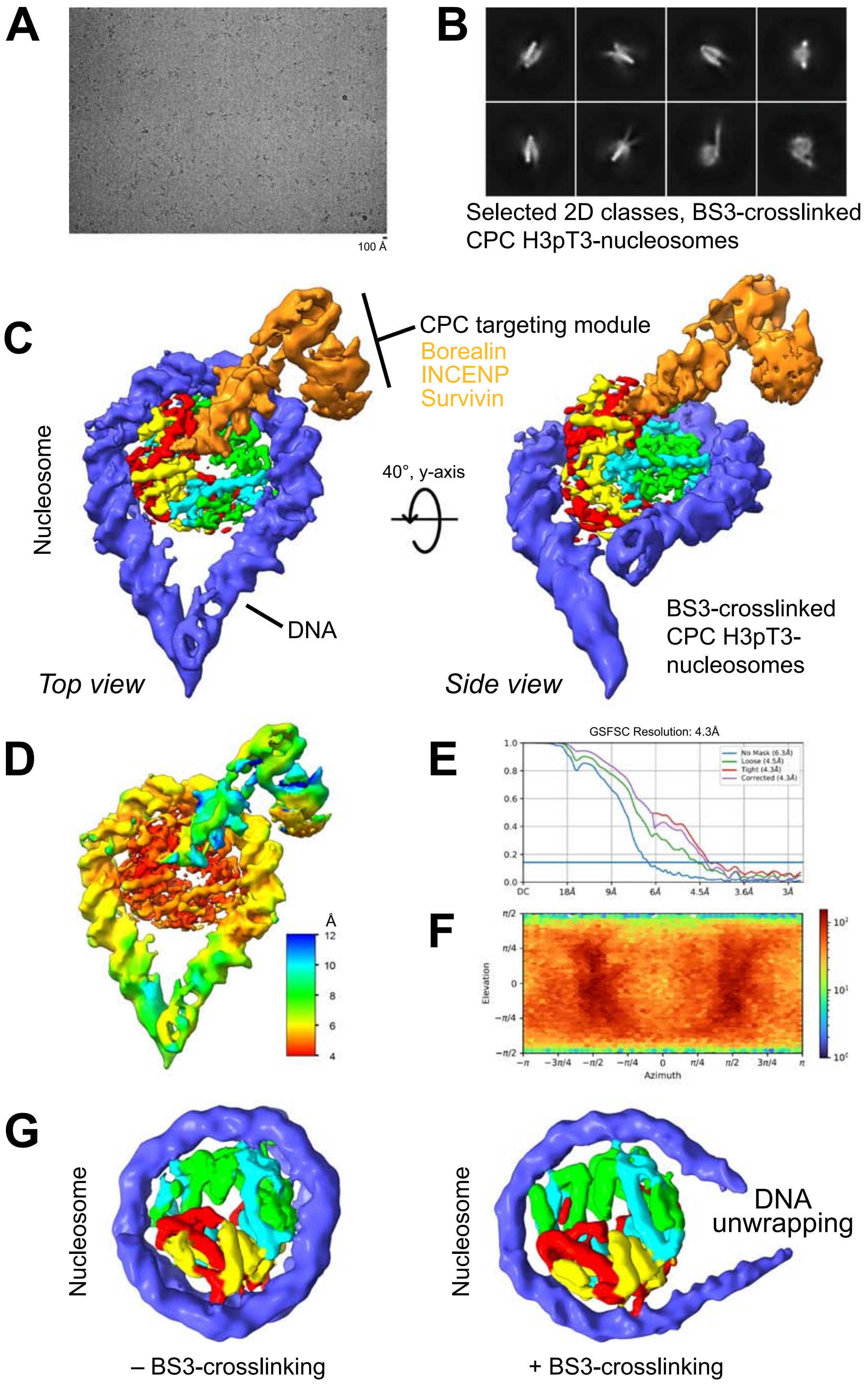
A
100 Å
B
Selected 2D classes, BS3-crosslinked
CPC H3pT3-nucleosomes
C
CPC targeting module
Borealin
INCENP
Survivin
Nucleosome
40°, y-axis
DNA
BS3-crosslinked
CPC H3pT3-
nucleosomes
Top view
Side view
D
Å
12
10
8
6
4
E
GSFSC Resolution: 4.3Å
No Mask (6.3Å)
Loose (4.5Å)
Tight (4.3Å)
Corrected (4.3Å)
DC
18Å
9Å
6Å
4.5Å
3.6Å
3Å
F
Elevation
Azimuth
G
Nucleosome
Nucleosome
DNA
unwrapping
– BS3-crosslinking
+ BS3-crosslinking

Figure EV3. BS3-crosslinked H3pT3-nucleosomes in complex with CPC80-Bor$_{(FL)}$.

(**A**) Example micrograph showing BS3-crosslinked CPC80-Bor$_{(FL)}$ H3pT3-nucleosome particles. (**B**) Selected BS3-crosslinked 2D classes. (**C**) Final volume for a CPC80-Bor$_{(FL)}$ H3pT3-nucleosome showing histone H2A (red), H2B (yellow), H3 (green), H4 (cyan), DNA (blue), and CPC subunit density (orange) in top and side view. (**D**) A local resolution map for the CPC80-Bor$_{(FL)}$ H3pT3-nucleosome complex. (**E**) Gold-standard Fourier shell correlation resolution (GSFSC), blue horizontal line denotes FSC value of 0.143, and (**F**) particle orientation distribution in the final map. (**G**) Models for H3pT3 nucleosomes analysed without (–) or with (+) BS3-crosslinking.

© The Author(s)

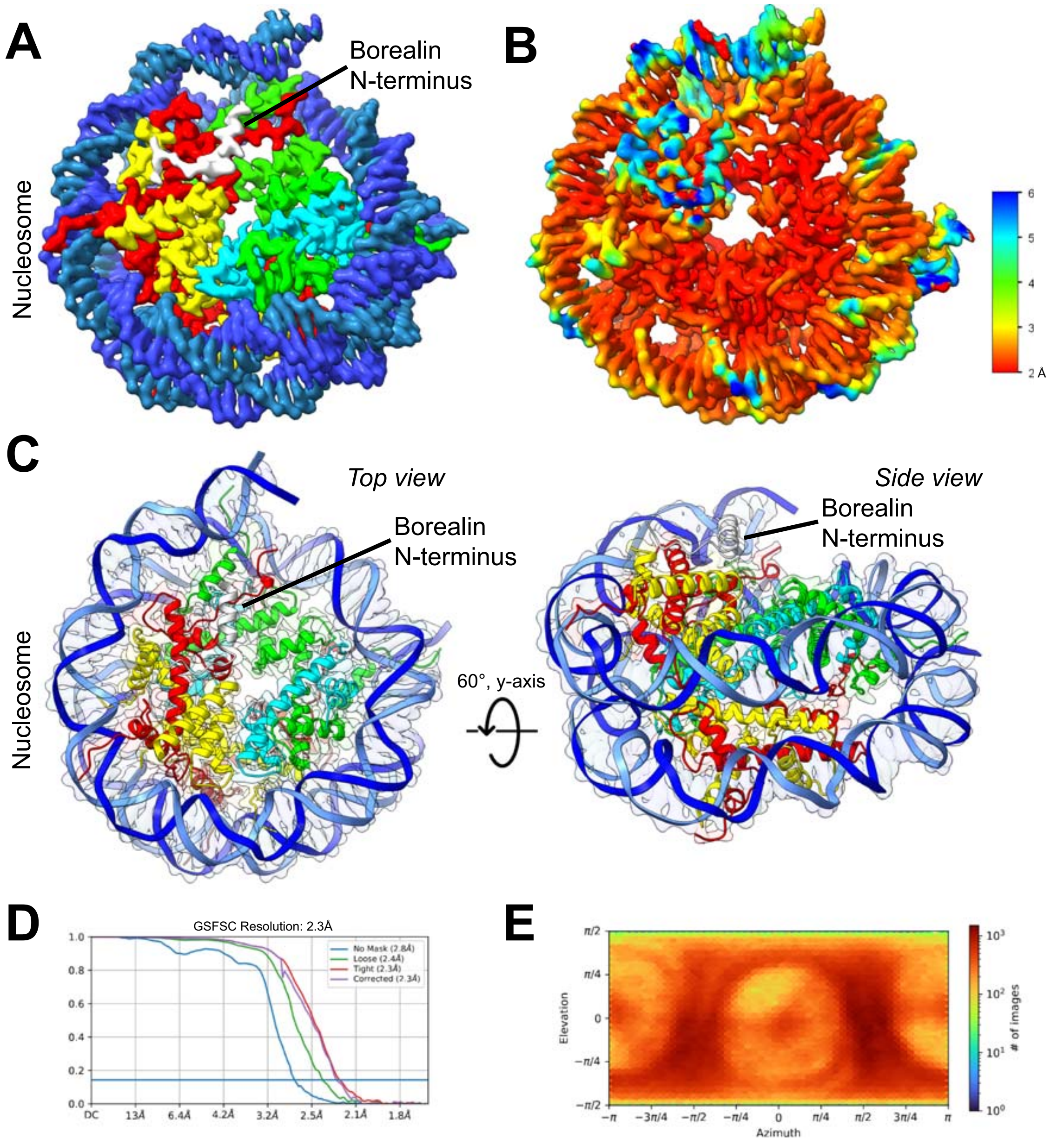

Figure EV4. Interaction of borealin with the nucleosome acidic patch.

(**A**) Cryo-EM density for the borealin N-terminus bound to a nucleosome showing histone H2A (red), H2B (yellow), H3 (green), H4 (cyan), DNA (blue), and borealin (grey/white), with (**B**) a colour-coded local resolution map (see inset scale 2–6 Å). PDB ID 8RUQ, EMDB ID - EMD-19514, Table EV1A (all particles). (**C**) Top (left) and side (right) views of the fitted model are shown. (**D**) Gold standard Fourier shell correlation (GSFSC) resolution, blue horizontal line indicates an FSC value of 0.143, and (**E**) particle orientation distribution within the final map.

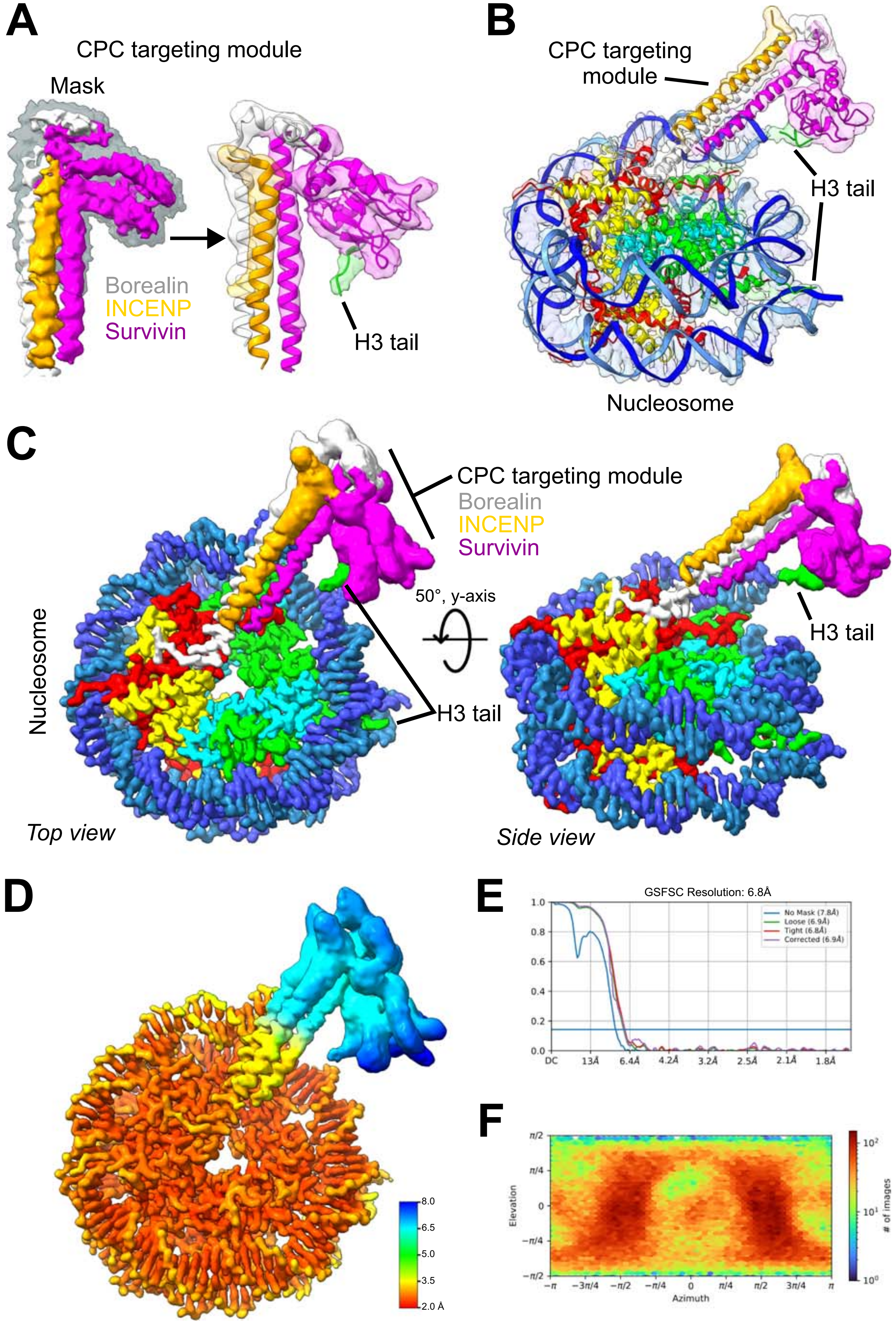
A
CPC targeting module
Mask
Borealin
INCENP
Survivin
H3 tail
B
CPC targeting module
H3 tail
Nucleosome
C
CPC targeting module
Borealin
INCENP
Survivin
Nucleosome
50°, y-axis
H3 tail
H3 tail
Top view
Side view
D
8.0
6.5
5.0
3.5
2.0 Å
E
GSFSC Resolution: 6.8Å
No Mask (7.8Å)
Loose (6.9Å)
Tight (6.8Å)
Corrected (6.9Å)
DC
13Å
6.4Å
4.2Å
3.2Å
2.5Å
2.1Å
1.8Å
F
Elevation
Azimuth
# of images

 © The Author(s)

Figure EV5. Interaction of survivin with the tail of histone H3.

(**A**) Local refinement of the CPC distal end with the masked region shaded in grey, with borealin (grey/white), INCENP (gold/orange), survivin (magenta), and histone H3 N-terminus (green). EMD-19684 and Table EV1C. (**B**) A fitted model for the CPC targeting module in complex with the H3pT3-nucleosome including H3 tail density associated with the survivin BIR domain. (**C**) Cryo-EM density of the CPC targeting module in complex with the H3pT3-nucleosome showing histone H2A (red), H2B (yellow), H3 (green); H4 (cyan), and CPC subunits borealin (grey/white), INCENP (gold/orange), and survivin (magenta). PDB ID 8RUP, EMDB ID–EMD-19513 and, Table EV1D (final). (**D**) A Local resolution map for the CPC targeting module in complex with the H3pT3-nucleosome including H3 tail density (green) associated with the survivin BIR domain. (**E**) Gold standard Fourier shell correlation (GSFSC) resolution, blue horizontal line indicates an FSC value of 0.143, and (**F**) particle orientation distribution within the final map.

© The Author(s)
